# Systematic identification of stem-loop containing sequence families in bacterial genomes

**DOI:** 10.1186/1471-2164-9-20

**Published:** 2008-01-17

**Authors:** Luca Cozzuto, Mauro Petrillo, Giustina Silvestro, Pier Paolo Di Nocera, Giovanni Paolella

**Affiliations:** 1 CEINGE Biotecnologie Avanzate scarl, Via Comunale Margherita 482, 80145 Napoli, Italy; 2 S.E.M.M. – European School of Molecular Medicine – Naples site, Italy; 3 DBBM Dipartimento di Biochimica e Biotecnologie Mediche, Universita' di Napoli FEDERICO II, Via S. Pansini 5, 80131 Napoli, Italy; 4 DBPCM Dipartimento di Biologia e Patologia Cellulare e Molecolare, Universita' di Napoli FEDERICO II. Via S. Pansini 5, 80131 Napoli, Italy

## Abstract

**Background:**

Analysis of non-coding sequences in several bacterial genomes brought to the identification of families of repeated sequences, able to fold as secondary structures. These sequences have often been claimed to be transcribed and fulfill a functional role. A previous systematic analysis of a representative set of 40 bacterial genomes produced a large collection of sequences, potentially able to fold as stem-loop structures (SLS). Computational analysis of these sequences was carried out by searching for families of repetitive nucleic acid elements sharing a common secondary structure.

**Results:**

The initial clustering procedure identified clusters of similar sequences in 29 genomes, corresponding to about 1% of the whole population. Sequences selected in this way have a substantially higher aptitude to fold into a stable secondary structure than the initial set. Removal of redundancies and regrouping of the selected sequences resulted in a final set of 92 families, defined by HMM analysis. 25 of them include all well-known SLS containing repeats and others reported in literature, but not analyzed in detail. The remaining 67 families have not been previously described. Two thirds of the families share a common predicted secondary structure and are located within intergenic regions.

**Conclusion:**

Systematic analysis of 40 bacterial genomes revealed a large number of repeated sequence families, including known and novel ones. Their predicted structure and genomic location suggest that, even in compact bacterial genomes, a relatively large fraction of the genome consists of non-protein-coding sequences, possibly functioning at the RNA level.

## Background

The availability of a massive amount of sequence data stimulated in-depth analyses on the organization of bacterial genomes [1-6]. Although less prominent than in eukaryotic genomes, sequence repeats are found in most bacterial species. According to their sizes, sequence repeats may be roughly classified into two main classes. Large repeats (0.8–2 kb) are mostly insertion sequences (IS), and encode proteins mediating their genomic mobility. The terminal inverted repeats (TIRs) and the nature of their gene products allow sorting ISs into specific classes [7,8]. Smaller repeats (50–300 bp) make up a much less defined and more variegate set of genomic sequences. Some of them contain palindromic sequences, demonstrated or proposed to be structured as stem-loops able to function as regulatory elements at DNA or RNA level. For example, *E. coli *PU-BIME elements have been shown to interact with the DNA gyrase [9] and the integration host factor protein [10], but also to function as mRNA stabilizers [11] and transcriptional attenuators [12]. Similarly, palindromic sequence repeats have been shown to function as mRNA stability determinants in *Neisseriae *[13-15] and *Yersiniae *[16,17].

Following these observations, and given the current availability of a large number of sequenced bacterial genomes, a systematic analysis of stem-loop containing repeated sequences appeared of interest. In a previous article [18], high stability stem-loop structures (SLS) were studied within a representative set of bacterial genomes and some of them were shown to have strong similarity with each other. Here we extend this study to detect all families of SLS containing sequences in the same bacterial set. To this aim, a pipeline, combining sequence clustering and Hidden Markov Model (HMM) based searches, was developed. This strategy led to the definition of a large number of sequence families, sharing sequence similarity and, in most cases, a common predicted secondary structure.

## Results

### Identification of initial SLS clusters

In a previous work a large number of SLS containing sequences were identified within 40 bacterial genomes [18]. For each bacterial species, sequences obtained from this study and predicted to fold with a free energy lower than -5 Kcal/mol were selected. In order to avoid obvious structured repeated sequences, they were filtered to eliminate those falling within either mature RNA species (tRNAs, rRNAs) or known ISs. An all-against-all BLAST comparison was performed on the selected sequences for the creation of a distance matrix, where distance is reported as the E-value of the found matches. Since SLSs are strand-specific, BLAST was run without searching for the complementary strand. Links between overlapping sequences were cut, by eliminating the corresponding matches from the matrix. The resulting matrix was then fed to a Markov Clustering algorithm (MCL) based tool [19] to produce a set of clusters. This clustering step was performed by using stringent parameters (see Methods) in order to favour the selection of more homogeneous clusters.

To avoid repeated analyses on the same genomic sequence, overlapping clustered SLSs were subsequently joined into larger SLS containing regions (SCRs). Clusters composed of at least 7 SCRs were selected and are reported in Table 1. Of the 40 analyzed genomes, 29 contain at least one cluster. The procedure led to the identification of 523 clusters, which together contain 28,904 elements, corresponding to 12,254 non-overlapping SCRs. No clusters were identified for the remaining 11 genomes: *L. innocua*, *L. monocytogenes*, *S. pyogenes*, *C. pneumoniae*, *C. trachomatis*, *U. urealyticum*, *R. prowazekii*, *T. pallidum*, *Buchnera*, *C. jejuni *and *H. pylori*.

**Table 1:** Sequence-based clustering of SLSs.

**Division**	**Species**	**SLSs**	**Clusters**	**Clustered SLSs**	**Clustered SCRs**
low-GC Firmicutes	*Bacillus anthracis*	65,220	4	105	38
	*Bacillus halodurans*	55,624	6	182	93
	*Bacillus subtilis*	56,622	2	32	16
	*Clostridium perfringens*	35,027	6	149	81
	*Clostridium tetani*	29,883	14	178	123
	*Enterococcus faecalis*	40,991	7	317	142
	*Lactobacillus johnsonii*	25,668	3	173	26
	*Staphylococcus aureus*	32,372	11	275	144
	*Streptococcus pneumoniae*	25,095	28	825	386
					
Mollicutes	*Mycoplasma genitalium*	8,953	1	21	8
	*Mycoplasma pneumoniae*	13,926	20	372	165
					
high-GC Firmicutes	*Corynebacterium diphtheriae*	54,254	9	282	120
	*Mycobacterium leprae*	83,094	29	1,721	537
	*Mycobacterium tuberculosis*	170,502	59	2,182	636
					
α-Proteobacteria	*Brucella melitensis*	69,899	11	399	219
	*Rickettsia conorii*	14,933	19	797	383
					
β-Proteobacteria	*Bordetella bronchiseptica*	214,459	26	2,009	470
	*Bordetella parapertussis*	188,237	30	1,513	518
	*Bordetella pertussis*	158,592	52	7,212	4,602
	*Neisseria meningitidis*	56,605	44	3,595	991
					
γ-Proteobacteria	*Escherichia coli*	86,339	12	1,152	431
	*Haemophilus influenzae*	25,055	3	39	25
	*Pasteurella multocida*	31,209	1	24	8
	*Pseudomonas aeruginosa*	206,492	9	526	129
	*Pseudomonas putida*	175,088	75	3,640	1,352
	*Salmonella typhi*	90,027	8	177	116
	*Salmonella typhimurium*	91,844	7	157	94
	*Vibrio cholerae*	45,824	7	250	122
	*Yersinia pestis*	78,372	20	600	279

**TOTAL**		**2,230,206**	**523**	**28,904**	**12,254**

BLAST-MCL clustering of SLSs identified, from a representative set of bacterial genomes, as described in Petrillo et al. [18]: only species with at least one cluster of a minimum of 7 elements are listed. For each species, the number of elements within the starting population, the number of clusters and the number of clustered SLSs are reported. The number of SLS containing regions (SCRs), obtained by fusing overlapping clustered SLSs, is also reported.

The clusters identified in each positive genome range between 1 and 75, for a total of 8 to over 4,000 clustered SCRs per genome. All together they correspond to about 1.3% of the originally selected population of over 2 million sequences. In order to evaluate the quality of the described clustering procedure, grouped SCRs were aligned by using the PCMA multiple alignment tool [20], and the resulting alignments were evaluated by ALISTAT [21]. Over 80% of the clusters showed an average identity better than 60%. The established consensus was larger than 90 bp for the about half of them, while the others produced consensus sequences between 27 and 90 bp (not shown).

### Clustered SLS containing sequences show high ability to form a stable secondary structure

The ability to fold into a reliable secondary structure was analyzed by using RANDFOLD [22], which compares the predicted minimum folding energy (MFE) of a sequence with those of a large number of random shuffles of the same sequence. Results are expressed as a p-value, representing the probability of the predicted MFE being truly different from the others. In this test, sequences were shuffled by preserving dinucleotide frequencies, as proposed by Workman and Krogh [23].

**Figure 1:**
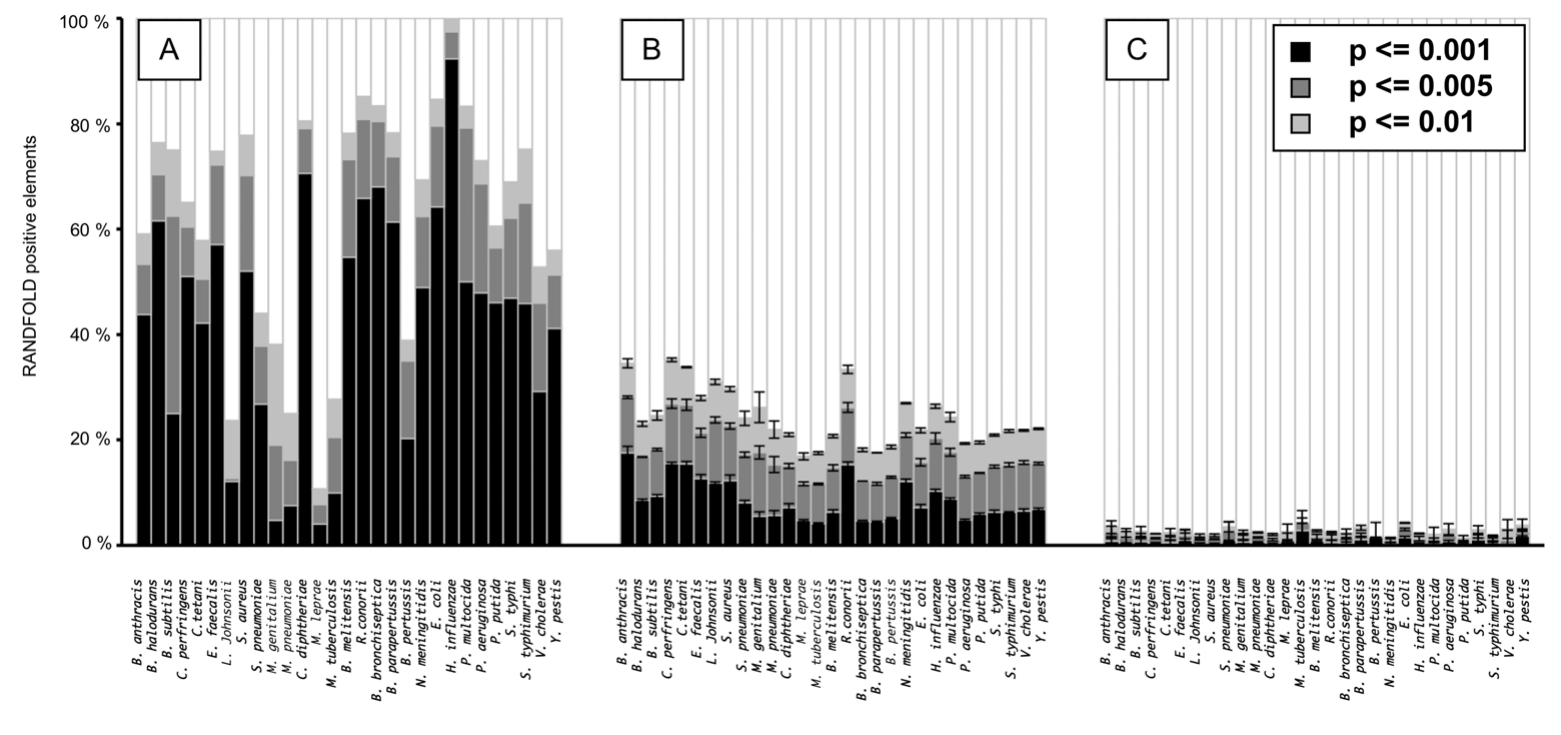
**Fraction of sequence elements positive to RANDFOLD test**. RANDFOLD test was run onto groups of clustered SLSs (panel A), total SLSs (panel B) and random sequences (panel C) from the 29 genomes listed in Table 1. The fraction of elements scoring positive with the indicated probability is diagrammed. Standard deviation bars are shown in panels B and C.

For each genome, the test was performed on clustered SLSs, as well as on SLSs randomly picked from the initial population and random sequences of equal size extracted from the same genome. The results are reported in Figure 1, where sequences are assigned to specific "folding aptitude" classes, according to the p-value calculated by RANDFOLD. Most clustered sequences (panel A) show a non-random probability of folding below 0.01 (dark grey bars), and, very often, also below 0.001 (black bars), whereas only about 20% of the original SLS population reach these p-values (Figure 1, panel B). Only for *M. leprae*, *L. johnsonii*, *M. genitalium *and *M. pneumoniae*, the two SLS populations do not show statistically different folding aptitudes. A very small fraction (less than 5%) of control sequences showed a non-random folding probability higher than 0.1% (light grey bars in Figure 1, panel C).

### Evaluation and refinement of the initial clustering

Various grouping procedures were used to combine the initial 523 clusters, according to sequence similarity, strand reciprocity and position on the genome. The results are reported in Table 2.

**Table 2:** Regrouping of SLS clusters.

		**Grouped by**			**Located within**	
		
**Species**	**Clusters**	**sequence**	**strand**	**location**	**IS**	**rRNA**
*B. anthracis*	4	3	2	2		
*B. halodurans*	6	6	4	3		1
*B. subtilis*	2	2	1	1		1
*C. perfringens*	6	2	1	1		
*C. tetani*	14	13	10	6	3	
*E. faecalis*	7	5	3	3	1	
*L. johnsonii*	3	3	2	2	1	
*S. aureus*	11	7	5	4		
*S. pneumoniae*	28	22	13	9	6	
						
*M. genitalium*	1	1	1	1		
*M. pneumoniae*	20	20	18	12		
						
*C. diphtheriae*	9	7	5	4	1	
*M. leprae*	29	18	11	5		
*M. tuberculosis*	59	36	21	15	3	
						
*B. melitensis*	11	7	5	4		
*R. conorii*	19	6	4	4		
						
*B. bronchiseptica*	26	8	5	4		
*B. parapertussis*	30	16	10	5	4	
*B. pertussis*	52	28	16	4	3	
*N. meningitidis*	44	9	7	6		
						
*E. coli*	12	8	6	6		2
*H. influenzae*	3	1	1	1		
*P. multocida*	1	1	1	1		
*P. aeruginosa*	9	5	4	4		
*P. putida*	75	35	26	14	4	2
*S. typhi*	8	4	3	3		2
*S. typhimurium*	7	6	4	4		1
*V. cholerae*	7	7	5	4		2
*Y. pestis*	20	15	11	5	2	

**Total**	**523**	**301**	**205**	**137**	**28**	**11**

Clusters reported in Table 1 were regrouped, according to sequence similarity, strand reciprocity and relative genomic position of their elements, as described in Methods. The number of groups, obtained by each criterion, is reported in the three columns labelled "Grouped by". Several groups are composed of sequences, contained within ISs or rRNA genes; their number is shown in the last two columns, for each genome.

In order to group clusters sharing sequence similarity, the clustered SCRs were re-clustered by using the above described BLAST-MCL based procedure, under less stringent conditions. The initial 523 clusters could be associated into 301 new clusters, most of them characterized by a larger number of elements (see column 'sequence' in Table 2). Within each new cluster, overlapping SCRs were fused as described above.

The ability to form SLS is generally shared by the two complementary strands of a given DNA sequence, the only exception being sequences where GU pairing is essential to form a stem-loop satisfying the minimum requirements. A number of clusters should therefore be composed of elements from the opposite strands of the same genomic region. Such clusters were identified, again by using the BLAST-MCL procedure, this time allowing BLAST searches also on the complementary strand. About two thirds of the clusters could be paired in this way, thus reducing the total number to 205 'unrelated' clusters (see column 'strand' in Table 2).

A third refinement was directed to connect clusters, which might represent different parts of a larger DNA repeat. For this reason, paired clusters, whose elements resulted to be overlapping or located at short distance (< 150 bp), were identified and joined within one group. This led to a further reduction to 137 cluster groups (see column 'location' in Table 2).

The resulting set was pruned by comparing SCRs from each cluster against the IS sequences collected in the ISfinder database [8] by using BLAST, in order to remove insertion sequences, possibly missed in the initial filtering. Similarly rRNA- and tRNA-related clusters were removed by evaluating the genomic localization of their elements, relative to genes encoding stable RNAs. These tests revealed that 28 cluster groups were related to insertion sequences, mainly not known at the time of the initial filtering, and 11 cluster groups were composed of sequence elements contained within rRNA precursors. These 39 cluster groups, reported in the columns 'IS' and 'rRNA' of Table 2, were flagged and not used for further analysis.

The whole procedure above described led to the identification of 98 candidate SLS-containing repeated DNA families.

### Characterization of families expanded by Hidden Markov Model searches

The candidate families were identified starting from small SLS containing sequences, which may be contained within regions of sequence similarity larger than the originally detected ones. In addition, genomic sequences may exist which, although similar, do not contain a SLS able to match the threshold used in the original search. For these reasons, a combined iterative procedure, based on HMM genome searches, was developed and applied to each family. In the procedure, a HMM built on the alignment of all family members is used to scan the parental genome and the detected sequences are aligned to the model. Alignments are extended by attaching neighboring sequences in order to define larger models, when possible. Multiple cycles of alignment, elongation, model building and genome search were performed until the borders of the repeated sequence were reached (see Methods).

A final, manual refinement was performed to combine essentially identical models. At the end of this procedure 92 models were obtained, which define the families reported in Table 3, where the length of the model and the number of detected sequences, both covering the entire model or part of it, are indicated. 67 models range in size between 31 and 200 bp, while the rest are larger, but only two extend over 1 Kb.

**Table 3:** Families of SLS containing repeated sequences.

*Species*	Family	This work		Literature			Type	Notes
		size	copies	size	copies	ref.		
*B. anthracis*	Bant-1	72	104(29)				I	
	Bcr1	167	31(21)	147	12	[24]	I	
*B. halodurans*	Bhal-1	74	36(32)				I	
	Bhal-2	76	50(41)				I	contains CRISPR repeats
*C. perfringens*	Clop-1	93	44(28)				I	
*C. tetani*	Clot-1	74	19(16)				I	
	Clot-2	31	34(32)					contains CRISPR repeats
	Clot-3	90	24(17)				I	contains CRISPR repeats
*E. faecalis*	Efa-1	163	65 (18)				I	
	Efa-2	292	11(9)				G	
*L. johnsonii*	Lac-1	231	34(6)				G	
*S. aureus*	Sta-1	105	25(25)				I	
	Sta-2	460	9(8)				S	
	Sta-3	136	24(15)				I	
	Sta-4	99	46(27)				I	
*S. pneumoniae*	BOX	84	205(105)	100–200	127	[25]	I	
	RUP	63	110(99)	108	54	[26]	I	
	Stre-1	45	241(225)				G	
*B. melithensis*	Bru-RS	118	222(69)	103–105	35–40	[27]	I	
*R. conorii*	Rpe-4	100	97(74)	95	94	[28]	I	
	Rpe-5	115	45(35)	115	55	[28]	I	
	Rpe-6	108	123(74)	136	168	[28]		
	Rpe-7	123	186 144)	99	223	[28]		
*M. genitalium*	Myg-1	259	10(7)				I	
*M. pneumoniae*	Myp-1	143	25(18)				G	part of REPMP1 repeat
	Myp-2	158	42(16)				G	part of REPMP4 repeat
	Myp-3	558	11(8)				G	part of REPMP5 repeat
	Myp-4	364	8(7)				G	part of REPMP5 repeat
	Myp-5	426	8 (8)				G	part of REPMP5 repeat
	Myp-6	468	11(11)				G	part of REPMP2/3 repeat
	Myp-8	674	9(9)				G	part of REPMP2/3 repeat
	Myp-9	226	9(9)				G	part of REPMP2/3 repeat
	Myp-10	330	12 (12)				G	part of REPMP2/3 repeat
	Myp-7	131	42(22)				G	
*C. diphtheriae*	Cod-1	140	17(16)				I	
	Cod-2	32	43(39)				G	
	Cod-3	170	23(20)					
	Cod-5	74	35(29)				I	
*M. tuberculosis*	Myt-1	72	75(70)					
	Myt-2	115	769(223)				G	located within PE genes
	Myt-3	81	81(77)				G	located within PE genes
	Myt-4	83	196(68)				G	located within PE genes
	Myt-5	71	41(2)				G	contains CRISPR repeats
	Myt-7	136	278(68)				G	located within PE genes
	Myt-8	92	33(25)					
	Myt-9	67	53(15)					
	Myt-10	154	62(59)				G	located within PE genes
	Myt-11	65	56(21)					contains MIRU repeats
*M. leprae*	REPLEP	740	29(9)	400–880	15	[29]	I	
	RLEP	641	38(30)	601–1075	37	[29]	S	
	Myl-1	371	7(4)				S	part of LEPREP repeat
	Myl-2	1979	9(7)				S	part of LEPREP repeat
*B. bronchiseptica*	Bor-1	117	196(92)				I	
	Bor-2	167	17(6)				I	
	Bor-3	134	34(32)				G	
	Bor-4	81	164(114)				G	
	Bor-5	112	135(101)				G	
	Bor-6	147	37(31)				G	
*B. pertussis*	Bor-1	93	128(78)				I	
*N. meningitidis*	ATR	206	14(9)	183	13	[30]	I	
	Nem-2	341	11(7)					
	Nem-3	127	10(9)				G	
	Nem-4	36	412(362)				I	contains DUS repeats
	dRS3	33	755(708)	20	770	[30]	I	
	NEMIS	46	262(81)	106–158	250	[13]	I	
	Rep2	65	22(18)	59–154	26	[30]	I	
*P. multocida*	Pam-1	155	12(12)				S	contains DUS repeats
*E. coli*	BoxC	50	22(20)	56	32	[31]		
	Eco-1	734	9(7)				G	
	ERIC	140	19(19)	127	21	[32]	S	
	PU-BIME	108	301(199)	40	485	[31]		
*H. influenzae*	Hin-1	31	53(51)				I	contains DUS repeats
*P. aeruginosa*	Pae-1	84	133(61)				I	
	Pae-2	287	65(24)				G	
	Pae-3	220	16(13)				G	
	Pae-4	52	41(35)					
*P. putida*	Ppu-1	617	39(28)				I	
	Ppu-2	2056	10(8)				S	
	Ppu-3	251	27(23)				G	
	Ppu-4	81	41(24)				I	
	Ppu-9	124	57(31)				I	
	REP	39	588(496)	30	804	[33]	I	
*S. typhi*	PU-BIME	43	146(126)	40	100	[31]	I	
	PU-BIME*	80	59(37)	40	>100	[31]		
*S. typhimurium*	PU-BIME	78	142(94)	40	82	[31]		
	Sal-1	115	27(17)				I	
	Sal-2	120	33(3)				G	contains CRISPR repeats
*V. cholerae*	ERIC	103	97(66)	127	80	[31]	I	
	Vic-1	184	14(1)				I	
*Y. pestis*	ERIC	115	241(128)	69–127	167	[16]	I	
	YPAL	168	101(68)	169	30	[17]	I	
	YPAL*	136	26(13)	130	10	[17]	I	

The final set of 92 families of repeated sequences is reported, grouped by species. For each family, the length of the model and the number of sequences fitting the model are given. The number of complete sequences, i.e. covering the model from end to end, is reported in parenthesis. Previously described sequence families have been named in column "Family", according to the current literature; for each of them, the number and typical size of its members are also provided, together with references. For novel families, a systematic name was built by fusing a shortened species name to a progressive number. In the column "type", I, G and S indicate the prevalent genomic location of the members of each families within intergenic, genic or border-spanning sequences. For some families, small previously described sequence motifs contribute to the formation of a substantially larger model; for others, their members are frequently located within larger previously described sequences. In both cases, a note is reported in the rightmost column.

BLAST comparison of all family elements, against the consensus sequences for DNA repeats described in literature, revealed that 25 families are already known, corresponding to essentially all previously identified SLS containing families. For each of them, size and copy number are reported in Table 3, along with the corresponding values derived from literature data [13,16,17,24-33].

The remaining 67 families are not described as such in literature. Their sizes range from 31 to over 2,000 bp for a number of elements varying between 9 and 164. Nine of these families (Bhal-2, Clot-2, Clot-3, Myt-5 Sal-2, Myt-11, Nem-4, Pam-1, Hin-1) contain known DNA sequence motifs, such as CRISPR [34], MIRU [35] and DUS [36]: the combination of two or more specific elements, matching these motifs, generates larger, SLS containing repeated sequences, not previously described. Sixteen families are made up of sequences contained within larger sequence blocks, either coding for abundant protein motifs or located within larger, ill-defined redundant intergenic sequences. Forty-two families appear to be unrelated to previously described sequence elements.

### Secondary structure analyses

Three different approaches were used to evaluate the aptitude of sequences from the detected families to fold into a common secondary structure (results are reported in Table 4):

**Table 4:** Secondary structure prediction analysis of families.

Species	Family	P	Conserved structure	Conserved SLS position	SLS folding aptitude	Type
*B. anthracis*	Bcr1	0.99	s	+	+	I
*B. halodurans*	Bhal-1	0.98	s	+	++	I
	Bhal-2	0.99	c		-	I
*C. perfringens*	Clop-1	0.96	s	+	+	I
*C. tetani*	Clot-1	0.95	s	+	++	I
*E. faecalis*	Efa-1	0.85	s	+	+++	I
	Efa-2	1.00	s	+	-	G
*L. johnsonii*	Lac-1	0.97	c	+°	-	G
*S. aureus*	Sta-1	0.84	s	+	+++	I
	Sta-2	1.00	s	+	++	S
	Sta-3	0.97	s	+	+	I
*B. melithensis*	Bru-RS	0.98	s	+	+	I
*R. conorii*	Rpe-4	0.73	s	+	-	I
	Rpe-5	1.00	s	+	+	I
	Rpe-6	0.45	-	+°	+	
	Rpe-7	0.99	s	+	++	
*M. genitalium*	Myg-1	0.06	-	+°	-	I
*M. pneumoniae*	Myp-1	0.00	-	+°	-	G
	Myp-2	0.95	s	+	++	G
	Myp-3	0.89	s	+	-	G
	Myp-4	0.09	-	+°	-	G
	Myp-5	0.74	s	+	-	G
	Myp-6	0.55	c		-	G
	Myp-7	0.67	s	+	-	G
*C. diphtheriae*	Cod-1	0.97	s	+	+++	I
	Cod-2	0.98	s		-	G
	Cod-3	0.99	s	+	+++	
*M. tuberculosis*	Myt-1	0.74	s	+	+++	
	Myt-8	0.90	s	+	++	
*M. leprae*	REPLEP	1.00	c	+°	-	I
	RLEP	1.00	s	+	++	S
	Myl-1	0.61	s	+	++	S
	Myl-2	0.97	s	+	+	S
*B. bronchiseptica*	Bor-1	0.86	s	+	++	I
	Bor-2	1.00	s	+	-	I
*B. pertussis*	Bor-1	0.93	s	+	++	I
*N. meningitides*	ATR	1.00	s	+	-	I
	Nem-2	0.93	s	+	+	
	Nem-4	0.93	s	+	+++	I
	dRS3	0.98	c		-	I
	NEMIS	1.00	s	+	+	I
	Rep2	0.98	s	+	+	I
*P. multocida*	Pam-1	0.96	s	+	+++	S
*E. coli*	BoxC	0.99	c	+°	-	
	Eco-1	0.18	-	+°	-	G
	ERIC	0.94	s	+	++	S
	PU-BIME	0.94	s	+	+	
*H. influenzae*	Hin-1	0.96	s	+	+	I
*P. aeruginosa*	Pae-1	0.97	s	+	++	I
	Pae-3	0.26	-	+°	-	G
	Pae-4	0.93	s	+	++	
*P. putida*	Ppu-1	0.97	s	+	+	I
	Ppu-2	1.00	s	+	+++	S
	Ppu-4	0.95	s	+	-	I
	Ppu-9	0.54	s	+	-	I
*S. typhi*	PU-BIME	0.97	c		-	I
	PU*-BIME	0.98	s	+	-	
*S. typhimurium*	PU-BIME	0.98	s	+	-	
	Sal-1	0.94	c		-	I
	Sal-2	1.00	c		-	G
*Y. pestis*	ERIC	0.90	s	+	-	I
	YPAL	1.00	s	+	+++	I
	YPAL*	0.96	c		-	I

The ability to form a consensus secondary structure was evaluated by RNAz: the prediction scores are reported in column "P" for each family. The type of predicted structure is indicated in column "conserved structure", where "s" indicates a stem-loop based structure, while "c" indicates a more complex structure, where a stem-loop compatible with the original search is not present. For each family, the aligned localization of the original SLSs is indicated by '+' in column "conserved SLS position"; when SLS alignment is not in agreement with the RNAz prediction, a '°' is added to the '+' symbol. The column marked "SLS folding aptitude" reports the behavior of family elements in the RANDFOLD test: the number of '+' symbols describes the percent of positive elements ('+++' if 90% or above; '++' if 70–90%; '+' if 50–70%; '-' if less than 50%). The localization of family members, as already described in Table 3, is also reported in the last column.

1) ability to form conserved secondary structures, evaluated, for each family, by RNAz [37] analysis of the alignment of six representative sequences to the family HMM (column "conserved structure");

2) presence of aligned SLSs and agreement with the structure predicted by RNAz (column "conserved SLS position");

3) probability of non-random folding for SLSs contained within each family, calculated by using RANDFOLD [22] (column "SLS folding aptitude").

Only families with either a predicted conserved secondary structure or aligned SLSs are reported. Of the 92 described families, 57 generate a common secondary structure, when analyzed by RNAz. For most (47) of them, marked as "s", the predicted structure contains a stem-loop compatible with the original search. In all but Cod-2, the position of the originally found SLSs is in agreement with the structure predicted by RNAz. These SLSs tend to be positive also to the RANDFOLD test: in 36 of the 47 families, most members contain SLSs, able to fold into a non-random secondary structure (P <= 0.005). For ten of the 57 families, indicated by "c", a more complex common structure is predicted by RNAz, not including a stem-loop compatible with the original search. Most of them, accordingly, do not feature aligned SLSs. Yet, the presence of aligned SLSs in three families (Lac-1, REPLEP, BoxC) may be seen as an indication for SLS-containing alternative folding.

RNAz failed to predict a common structure for 35 of the 92 families: for most of these families (29 out of 35) no aligned SLSs are available, indicating the absence of common secondary structures. Aligned SLSs are present in 6 families (Myg-1, Myp-1, Myp-4, Eco-1, Pae-3, RPE-6), which score negative at the RNAz test: for all but RPE-6, aligned SLSs show a low folding aptitude (see Table 4).

### Genomic localization

Genomic localization of the families is reported in Table 3 where, in column "type", families are classified, according to the position of the vast majority of their members, relative to annotated coding sequences. 41 families are intergenic (I), 30 genic (G) and 7 tend to span the borders between coding and non coding sequences, and are therefore indicated as border spanning (S).

For 14 families no clear predominance of genic or intergenic sequences was observed, and therefore the family was not assigned to a class.

Genomic localization of the families predicted to fold in a secondary structure is reported in Table 4; for all families, genomic localization, correlated with the predicted ability of the family members to fold into a common, stable secondary structure, is summarized in Table 5. For most intergenic families a secondary structure is predicted (31 out of 41). Genic families, in contrast, are predominantly not structured: only about one third (9 out of 30) have a structure predicted by RNAz and only for 5 of them aligned SLSs support its existence. Border spanning and unclassified sequence families feature a predicted secondary structure with frequencies similar to intergenic ones.

**Table 5:** Structural properties of the described SLS families in relation to genomic location.

	Sec. Struct. +		Sec. Struct. -		**Total**
		
Genomic location	SLS +	SLS -	SLS +	SLS -	
Genic	5	4	4	17	**30**
Border spanning	7	0	0	0	**7**
Intergenic	25	6	1	9	**41**
Others	9	1	1	3	**14**

**Total**	**46**	**11**	**6**	**29**	**92**

Columns under "Sec. Struct. +/-" report the number of families, characterized by the presence or absence of a conserved secondary structure predicted by RNAz; the labels "SLS +/-" indicate the presence or absence of aligned SLSs; "Total" means the sum of rows or columns.

### Characterization of specific families

**Figure 2:**
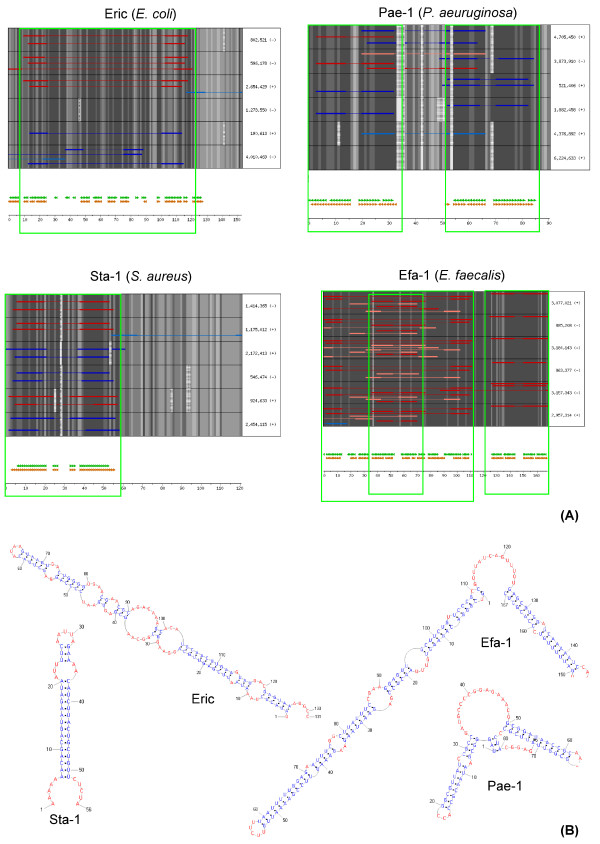
**Alignment of ERIC, Pae-1, Sta-1 and Efa-1 family members**. **(A) **A representative set elements from each family was aligned by using the HMM model as a guide. In each panel, one row corresponds to one family member (indicated on the right with its genomic position). Within each row, sequence conservation is indicated by increasing gray levels and gaps by dotted spaces; overlapping SLSs are reported as red and blue lines, the red ones indicating SLSs used to define the original HMM model for the family, the blue all the others. Darker colors indicate the SLS folding aptitude, i.e. positivity to RANDFOLD for P <= 0.005. Common secondary structures, predicted by RNAz, are reported at the bottom, just above the ruler in nucleotides: green triangles indicate stems produced by pairing complementary regions on the same strand as the identified SLSs, while brown triangles indicate the same from the opposite strand. The boxed regions highlight areas where aligned SLSs and predicted structures are in agreement. **(B) **Graphic representation of the RNAz predicted secondary structures.

The described procedure led to the identification of a large number of families of repeated bacterial sequences, some already known, other previously undescribed. For many of them, a number of tests showed the potential folding of the majority of their members into a shared secondary structure. Four such families are reported in Figure 2, where the predicted secondary structure is shown along with the aligned, originally found, SLSs. One of them, the ERIC family from *E. coli*, had previously been described, while the other three are new. ERIC, as anticipated from literature reports [31,32], is predicted to fold into a single, long stem-loop structure. Sta-1 folds into a simple, shorter SLS. Pae-1 and Efa-1 families feature more complex structures, composed of a pair of adjacent SLSs. The structures predicted for these four families may be predicted on both strands, with complementary sequences generally, but not necessarily, folding into corresponding stems. For Pae-1, the prediction of different structures on the two strands indicates the likely presence of multiple foldings of comparable stability, which, on each strand, are alternatively selected as the best one, because of minor base pair differences.

**Figure 3:**
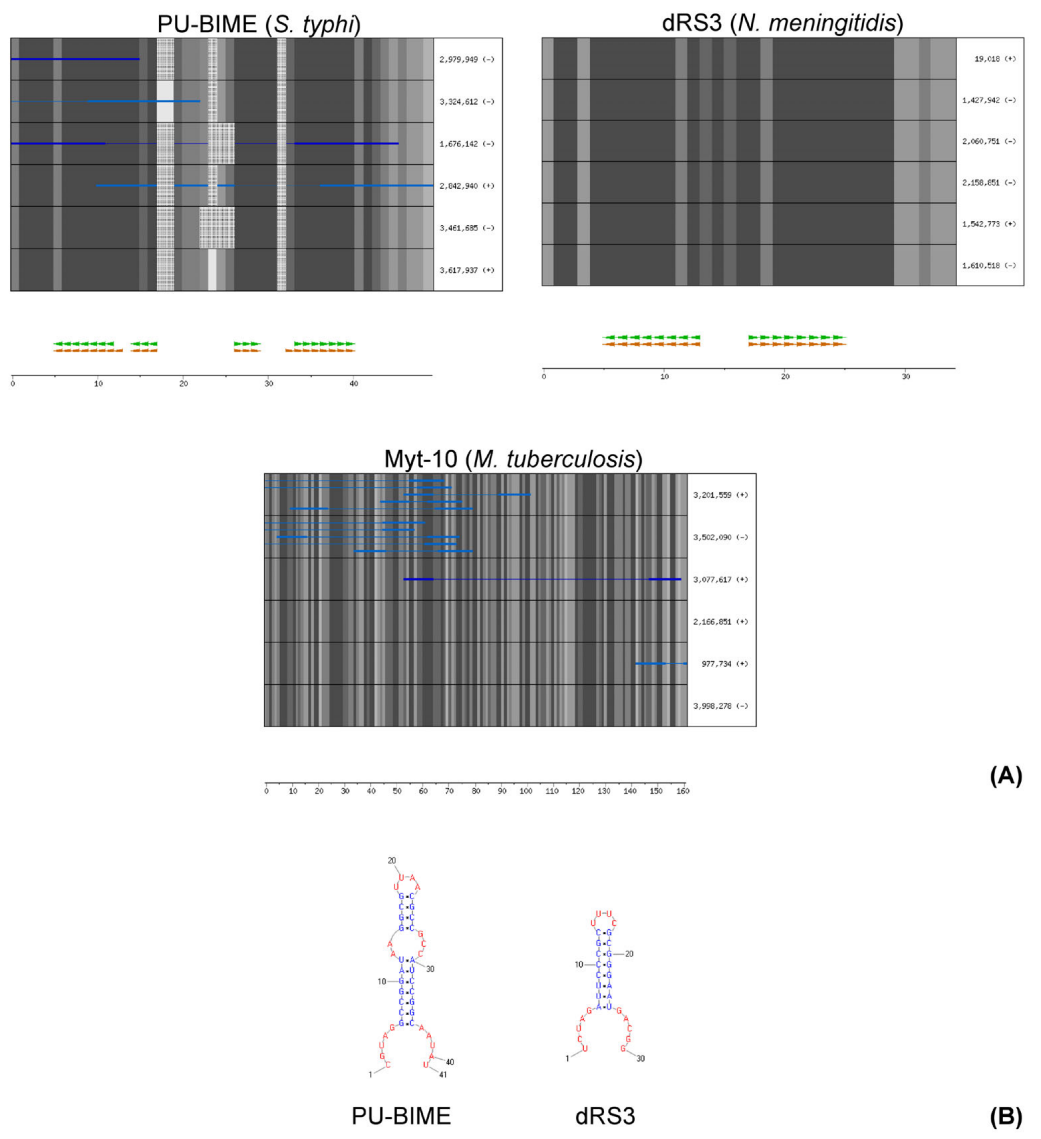
**Alignment of PU-BIME, dRS3 and Myt-10 family members**. Panels **A **and **B **legends are as in Figure 2.

For some of the identified families, secondary structure predictions, although supported by high RNAz scores, are not consistent with the originally found SLSs. Generally this stems from the prediction, by RNAz, of structures not including SLSs fitting with the original SLS definition. PU-BIME and dRS3, shown in Figure 3, are examples of such families: in PU-BIME the stem includes a five base internal loop, while in dRS3 the 8 bp stem is too short. Both cases are not compatible with the original search (see Methods).

Finally, for about one third of the 92 identified families, it is unlikely that a RNA secondary structure may play a relevant role, as shown by the absence of either a common predicted structure or alignment of originally found SLSs. An example of such families is Myt-10, reported in Figure 3.

## Discussion

In a previous study, a systematic analysis of putative SLSs found in bacterial genomes showed that they tend to be more abundant and stable than those randomly formed in shuffled sequences of comparable size and base composition [18]. This observation led to the hypothesis that, along with SLSs stochastically formed because of sequence composition, a sizeable quota is possibly the result of selective pressure, due to the need to preserve a biological function. SLS-containing secondary structures are known to play a relevant role in several aspects of gene expression and its regulation. Structured RNAs are a functional component of enzymes like RNAse P [38], or contribute to the formation of regulatory cis-acting regions such as riboswitches [39], thermosensors [40], transcriptional attenuators and terminators [41,42]. Palindromic RNA sequence repeats may also influence mRNA stability [11].

**Figure 4:**
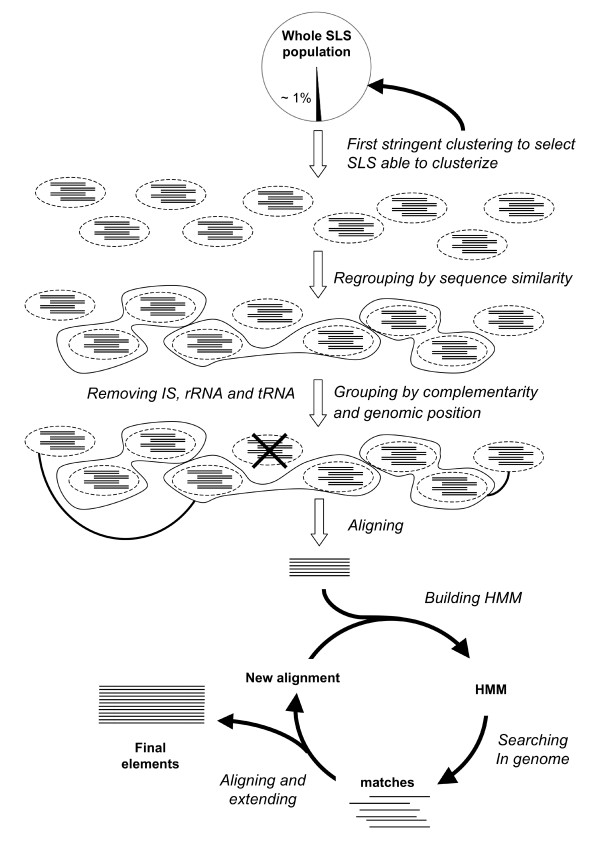
Schematic representation of the overall procedure.

In this work, we describe a systematic procedure, schematically depicted in Figure 4, to identify and classify families of repeated sequences, characterized by a shared secondary structure, in the genomes of a representative set of bacteria, most of which of medical interest. To this aim, SLS containing sequences were first clustered by sequence similarity and subsequently evaluated for their potential to form secondary structures. In most analyzed genomes, a fraction of SLSs could be grouped into clusters, containing at least 7 non-overlapping elements. No clusters were found in 11 of the 40 analyzed genomes.

Clustering by sequence similarity resulted in selection of 523 clusters corresponding to just above 28,000 SLSs, about 1% of the whole SLS population: this figure may vary quite a lot in specific species, being sensibly larger, up to 6%, in *N. meningitidis*, and substantially lower in *B. subtilis *and *P. multocida*, where less than 0.1% of the SLSs fall within clusters. Clustering ended up by selecting a subset of SLSs different from the original population and characterized by a much higher probability of non-random folding (see Figure 1), indicating that selection based on sequence similarity was very effective in enriching for structured regions.

Various refinement steps produced the final set of 137 clusters, reported in Table 2. Although mature rRNA and tRNA genes were initially masked within the searched genomic sequences, some clusters were identified, which correspond to unmasked parts of ribosomal RNA precursor genes (Table 2). Similarly, some clusters correspond to SLSs contained within ISs, which escaped the initial filtering for various reasons. Removal of these two subsets and other redundancies reduced the number of identified families to 92.

Notwithstanding the starting population of SLS containing sequences, within these families regions sharing primary structure similarity, but not a common SLS, might, in principle, still be found, and 35 families with no recognizable shared secondary structure, were indeed identified. Most of these sequences are, not surprisingly, found within coding regions, where the formation of secondary structures is expected to be limited by the translation machinery. However, some of these families coincide with intergenic sequence repeats, such as the *S. pneumoniae *BOX and *P. putida *REP sequences unable to form structures compatible with the originally searched ones.

### Families sharing common secondary structures

Most identified families, 57 out of 92, are predicted by RNAz to share a common secondary structure. This group includes well-known intergenic families, such as the *E. coli *PU-BIME and ERIC repeats, and their homologues in other species, as well as a number of less known families, most of which described in isolated reports, but not characterized in detail (see Table 3). Practically all intergenic repeats, previously shown or predicted to fold into a RNA secondary structure, have been found. The only exceptions are the *S. pneumoniae *RUP and the *R. conorii *RPE-6 repeats, which, although identified by the pipeline, do not fall into this group, because RNAz could not predict a shared secondary structure better than the defined threshold.

For known families, the sequence boundaries, as predicted by the pipeline, are essentially coincident with those previously reported in literature. Specific discrepancies were found only in two families. In the *N. meningitidis *NEMIS elements, the present search identified the central 46 bp core, but failed to extend the similarity to either the partial 108 or the complete 158 bp repeats described by Mazzone et al. [13]. Similarly, for the *S. pneumoniae *RUP family, only 63 bases were detected out of the complete 108 bp elements [26].

### Known and novel families

In well characterized genomes, such as those of enterobacteria, practically all known families have been detected, along with a few new ones. In *E. coli*, the known PU-BIME, ERIC and BoxC families were recognized and feature shared secondary structures, while the only new one identified, the Eco-1 family, is predicted as unable to fold. PU-BIME repeats were also detected in *S. typhi *as two related variants (a full size and a shorter one, only the former predicted to fold) and in *S. typhimurium*, along with two novel families, Sal-1 and Sal-2 (Table 3). For both of them RNAz could predict a shared secondary structure of the complex type.

As expected, ERIC sequences were detected not only in *E. coli*, but also in *Y. pestis *and *V. cholerae *[16,31]: *Y. pestis *repeats are predicted to fold with a structure closely similar to the *E. coli *elements. In contrast, ERIC sequences detected in *V. cholerae *are not predicted to fold, being 20 bp shorter than both *E. coli *and *Y. pestis *homologues, because of selective erosion of their TIRs. *Yersiniae *ERIC sequences have been shown to regulate the level of expression of neighboring genes by folding into RNA harpins [16]. *V. cholerae *ERIC, being unable to fold, may thus not function as RNA stability determinants.

Most potentially structured new families have been found in species less analyzed experimentally or whose genome was more recently sequenced, such as pseudomonaceae, bordetellae, mycobacteria.

For both novel and known families, the predicted common secondary structure is often a stem-loop (see Sta-1 and ERIC in Figure 2). In a fraction of cases, however, RNAz analysis proposes different structures. Some families feature a double hairpin (see EFA-1 and Pae-1 in Figure 2) and others feature a complex structure containing a SLS (not shown).

### Genomic localization

Genomic localization highlights the preferential tendency of repeated sequences with a predicted common secondary structure to lie within intergenic regions; this is true for both known and novel ones. In contrast, families found within coding sequences (CDSs) of genomes are often not structured. This is in agreement with the results of RANDFOLD analysis: most (19 out of 27) intergenic families with aligned SLSs (Table 4) are enriched in highly structured SLSs, while this is true for only one genic family, Myp-2. These observations support the overall hypothesis that many of these sequence families fold in a secondary structure at the RNA level, particularly those located in intergenic regions, where the translation machinery is not expected to interfere with secondary structure formation.

Three novel intergenic structured families, Hin-1 in *H. influenzae*, Nem-4 in *N. meningitidis *and Pam-1 in *P. multocida *are composed of similar sequences, characterized by the repetition of short, abundant oligonucleotides, known as DUS [36]. The recurrence, at specific short distances, of this basic oligonucleotide module, shorter than the searched pattern, produces a conserved SLS larger than the required threshold. It is possible that these sequences function as transcriptional terminators, and it has been recently reported that terminator hairpins are indeed frequently formed by closely spaced, complementary instances of exogenous DNA uptake signal sequences [43].

Some novel structured families are located within CDSs. They often contain repetitive motifs of one or a few coding regions, such as Lac-1 in *L. johnsonii*, Pae-3 in *P. aeruginosa *and Efa-2 in *E. faecalis*. Interestingly, the Cod-2 family defines a very small repeat, found within various CDSs, encoding different peptides in different frames. Cod-2 repeats resemble repetitive sequence elements found by Claverie and coworkers in protein coding genes of *R. conorii *[44]. Five genic families found in *M. pneumoniae *are part of large (1.5–5.4 kb), possibly mobile repeated DNA sequences having coding capacity [45].

About one third of the identified families are found to be "unstructured". These sequences were not the object of the original search; a possible explanation of their detection is the incidental presence of SLSs within large repeated sequences. Most such families fall within CDSs (see Table 4, and Myt-10 in Figure 3 as an example). Ten of them are contributed by only two genomes: *M. tuberculosis *and *M. pneumoniae*. Other unstructured families are clustered within the same CDS (Bor-3 and Bor-6 in *B. bronchiseptica*) or are dispersed within multiple CDSs, sharing a common protein domain (Bor-4 and Bor-5 in *B. bronchiseptica*, Pae-2 and Ppu-3 in *P. aeruginosa *and *P. putida*, respectively).

## Conclusion

A systematic analysis of 40 bacterial genomes is presented, aimed to identify repeated sequence families, sharing a common predicted secondary structure. This procedure identified practically all already described families meeting these constraints, as well as a larger number of novel, undescribed nucleic acid repeats.

About two thirds of the families shared a predicted conserved secondary structure, often a stem-loop based one. Interestingly, these families are mostly composed by elements located within intergenic regions. This localization reflects the hypothesis that RNA folding, within these regions, is more likely to occur, not being affected by the translation machinery.

The identification of repetitive sequence families, able to fold into secondary structures and preferentially located within intergenic regions, reinforces the notion that also in prokaryotic genomes, typically more compact than eukaryotic ones, a relatively large fraction, not coding for proteins, is likely to play a biological role, by encoding functional RNAs.

## Methods

### Selection of SLS clusters

SLSs previously identified in 40 bacterial genomes by Petrillo at al. [18] were taken as the starting population. Only SLSs predicted to fold with a free energy <=-5 Kcal/mol were used for the present study.

For each genome, selected SLSs were clustered according to a procedure, based on BLAST and MCL programs [46,19]. An all-against-all BLAST comparison was performed on the whole population, to create an E-value based distance matrix. The BLAST result matrix was pruned by removing hits linking overlapping SLSs, and subsequently fed to MCL to produce a set of clusters. BLAST was performed with an E-value cut-off of 1E-4 and only on the sequence top strand. MCL was run by setting the inflation parameter (I)equal to 4. The alignments of clustered elements were produced by PCMA [20] used with default parameters.

### Aptitude to form a stable secondary structure

The aptitude of SLSs and control sequences to form a stable secondary structure was tested by running RANDFOLD [22]. The '-d' option was used, in order to preserve dinucleotide frequencies. RANDFOLD was set to shuffle each sequence 1,000 times. In the tests reported in Figure 1, all clustered SLSs (panel A) were compared to a number of SLSs representing the 5% of initial SLS population (panel B) and to a number of genomic sequences having the same size of clustered SLSs, randomly extracted from the corresponding genomes (panel C). Control sequences analyzed in panels B and C, were selected three times, in order to evaluate average and standard deviations.

### Cluster refinement

The regrouping procedures summarized in Table 2 were made as follows:

• **Regrouping by sequence **was made by using the BLAST-MCL procedure (see above) on all SCRs, but in a less stringent way, i.e. setting parameter 'I' to 1.4.

• **Regrouping by strand **was performed by using the BLAST-MCL procedure, but allowing searches on the complementary strand and setting parameter 'I' to 1.4.

• **Regrouping by location **was obtained by merging clusters in which SCRs were partially overlapping, or within a distance of 150 bp, according to their genomic coordinates.

For each regrouping procedure, groups of clusters, sharing at least 50% of the elements, were fused into a larger one.

### Identification of families by cycles of HMM searches

In order to identify all family members of each cluster, a procedure was developed, based on cycles of alignment by PCMA and search on the genome by HMMER package tools [21]. First, SCRs of clusters regrouped by sequence (see Table 2) were aligned by PCMA with option 'ave_grp_id' set to 50. The procedure can be summarized as it follows:

1. The alignment is used to build a HMM by HMMBUILD and HMMCALIBRATE, with the default options.

2. The produced HMM is used to search new elements within the genome, by using HMMSEARCH. E-value cut-off was set to 1E-10. Independent searches are run on each genomic sequence strand.

3. Identified sequences are extracted and aligned to their parental HMM by HMMALIGN. Pairs of overlapping sequences on the opposite strands are avoided by discarding the one with the worse score and E-value.

4. The aligned sequences are extended by 10% of the length of the parental HMM. Only the extensions are aligned by PCMA.

5. The alignment of the extended sequences is then used for the construction of a new model, returning to step 1.

The loop ends when one of the following criteria is met:

• The detected sequences, which cover the entire model, are less than 7.

• The new model is shorter in terms of length than the previous one.

• The alignment does not extend the HMM any further (within a tolerance of 3 bp).

• The alignment contains a number of gaps higher than 30% of the aligned bases.

• The extreme value distribution, derived from the model calibration, is in the range Average_Score ± 3*Standard_Deviation, derived from HMMBUILD.

The HMM and the final alignment are used as definition of the family.

### Secondary structure analyses

SLSs contained in sequences of each family were analyzed by RANDFOLD as described above and taken as positive if their p-value is < 0.005. Families were divided in four categories, according to the fraction of sequences containing at least one positive SLS ('+++' if 90% or above; '++' if 70–90%; '+' if 50–70%; '-' if less than 50%).

Representative sequences of the families shown in Figures 2 and 3 were chosen in the following way:

• All sequences able to cover the entire model are sorted by the E-value determined by HMMSEARCH.

• Six sequences are picked from this population by selecting the best model-fitting one and five (if available) more with progressively increasing of the E-value.

Sequences were aligned to corresponding HMM by using HMMALIGN and the resulting alignments were analyzed by RNAz (version 0.1.1) [37]. For RNAz analysis, alignments with length <= 200 bp were used as a single block, while alignments with length >200 bp were screened in sliding windows (length 120 and slide 40), according to Washietl et al. [47].

RNAz was used with standard parameters. All alignments with RNAz classification score P > 0.5 were considered. Overlapping hits, i.e. resulting from hits in overlapping windows, were analyzed again by using larger sliding windows able to contain structures obtained with different hits.

## Abbreviations used

bp: Base pair; CDS: Coding sequence; CRISPR: Clustered regularly interspaced short palindromic repeats; DUS: DNA uptake sequence; HMM: Hidden Markov Model; IS: Insertion sequence; MCL: Markov Clustering algorithm; MFE: Minimum folding energy; MIRU: Mycobacterial interspersed repeated unit; nt: nucleotide; SCR: SLS-containing region; SLS: Stem-loop-structure; TIR: Terminal inverted repeat.

## Conflicts of interests

The author(s) declare that they have no competing interests.

## Authors' contributions

LC and MP designed the procedure for clustering of SLSs into families. They also analyzed sequence family members, respect to folding aptitude and secondary structure prediction. GS retrieved the literature information and provided manual annotation and analysis. PPDN and GP conceived and coordinated the study. All authors read and approved the final manuscript.

## Supplementary Material

Additional file 1 Family sequences. A zipped archive of files corresponding to families described in Table 3. Each file contains all the family members as sequences in FASTA format. Click here for file
